# Before and after enforcement of GDPR: Personal data protection requests received by Croatian Personal Data Protection Agency from academic and research institutions

**DOI:** 10.11613/BM.2020.030201

**Published:** 2020-08-05

**Authors:** Livia Puljak, Anamarija Mladinić, Ron Iphofen, Zvonimir Koporc

**Affiliations:** 1 Center for Evidence-Based Medicine and Health Care, Catholic University of Croatia, Zagreb, Croatia; 2 Croatian Personal Data Protection Agency (AZOP), Zagreb, Croatia; 3 Independent Research Consultant, La Rochelle, France

**Keywords:** data protection, scientific research, European data protection board, general data protection regulation

## Abstract

**Introduction:**

The European Union’s (EU) General Data Protection Regulation (GDPR) was put in force on 25th May 2018. It is not known how many personal data protection requests the national authority in Croatia had received before and after GDPR, and how many of those were related to research.

**Materials and methods:**

We obtained data from the Croatian Personal Data Protection Agency (CPDPA) about requests/complaints related to personal data protection that were received specifically from academic/research institutions, specifically the number and type of all cases/requests between the years 2015-2019.

**Results:**

In 2018, CPDPA had a dramatic increase in the number of requests in the post-GDPR period, compared to the pre-GDPR period of the same year. In 2019, CPDPA received 2718 requests/complaints; less than in the year 2018. From 2015 to 2019, CPDPA received only 37 requests related to research.

**Conclusions:**

Very few requests about personal data protection from academic and research institutions in Croatia were submitted to the national Croatian data protection authority. Future studies could explore whether researchers have sufficient awareness and knowledge about personal data protection related to research, to adequately implement the GDPR regulations.

## Introduction

Emerging new technologies are triggering novel ethical questions related to data protection and privacy (*1*). In 2002, Schermerhorn defined ethics as the „code of moral principles that sets standards of good or bad, right or wrong, in one’s conduct and thereby guides the behaviour of a person or group“ (*2*). When it comes to data protection and privacy, the European Union’s (EU) General Data Protection Regulation (GDPR) has an important role (*1*).

The GDPR, enforced on 25 May 2018, has replaced the EU’s previous legal framework on data privacy regulation – a directive – that had been in operation from 1995 (*3*). While it has retained the overall regulatory approach, the GDPR has also introduced multiple new compliance obligations, including greater sanctions, compared to the previous legal framework (*4*).

The idea behind the GDPR was to better regulate and safeguard personal data protection and privacy. Additionally, GDPR also aims to facilitate and alleviate the processing of personal data for scientific and research purposes by providing exemptions for scientific research. Its article 89 mentions pseudonymization as one of the measures to ensure the respect for the principle of data minimization. In addition, article 89 specifically states that: “*Union or Member State law may provide for derogations from the rights referred to in Articles 15, 16, 18 and 21 subject to the conditions and safeguards referred to in paragraph 1 of this Article in so far as such rights are likely to render impossible or seriously impair the achievement of the specific purposes, and such derogations are necessary for the fulfilment of those purposes.*” (*3*).

In Croatia, the Act on the Implemantation of the General Data Protection Regulation (Official Gazette, No. 44/2018) was enacted on 25th May 2018 to ensure full implementation of the General Data Protection Regulation, but derogations for scientific research purposes at the national level were not implemented, except for the official Croatian statistical purposes (Article 33) (*5*). This lack of clarity made science and research in Croatia even more demanding from the perspective of personal data protection.

General Data Protection Regulation regulates issues of a data breach, indicating that a data breach could result in physical, material or non-material damage, and specifies that when a data controller becomes aware of a personal data breach, the controller needs to notify the supervisory authority within 72 hours after finding out about it (*3*).

As a supervisory national authority, the Croatian Personal Data Protection Agency (CPDPA) is a member of the European Data Protection Board (EDPB), and representatives of CPDPA participate regularly in the work and activities of EDPB and its expert subgroups in order to be involved in development of guidelines and opinions and to keep pace with the latest development in the data protection field (*6*).

Currently, there are no publicly available data about the data protection needs, requests and data breach cases from academic and research institutions in Croatia that would require consultations with CPDPA and potentially further actions such as legal actions.

The primary aim of this current study was to analyse the number, type and outcomes of data protection requests that were submitted by academic/research institutions in Croatia to CPDPA before and after enforcement of GDPR. A secondary aim was to analyse the number and outcome of all requests about personal data protection that were submitted to CPDPA immediately before and after GDPR enforcement.

## Materials and methods

### Terminology

In this manuscript we used terminology as defined in the GDPR (*3*). *Personal data* refers to any information identifying natural person, *i.e.* ‘data subject’ (*3*). *Pseudonymisation* is a process which ensures that personal data are processed in a way that prevents them being attributed to a specific data subject (*3*). *Data subject* is a natural person whose data are being processed. *Data controller* is the body which: “*determines the purposes and means of the processing of personal data*” (*3*). *Data processor* is the body that: “processes personal data on behalf of the controller” (*3*). *Requests/legal advice* – from data subjects (citizens) who ask for information about their rights, and data controllers and processors who ask for information about their obligations and advices how to comply with the data protection legal framework. *Complaints*, *i.e.* requests for determination of a violation of a right from data subjects (citizens); anyone who considers that any of his or her rights guaranteed by the GDPR and the Act on the implementation of the GDPR have been violated, may submit to the Agency a request for determination of a violation of a right. *Administrative procedures* are initiated by the CPDPA if the request for determination of a violation of a right (complaint) is valid. The General Administrative Procedure Act (Official Gazette, No. 47/2009) as a general procedural act regulating rules of procedure in administrative matters shall apply to administrative proceedings pending before CPDPA. Parties with contrary interests are involved in the administrative proceedings before CPDPA and for these reasons an investigation is being conducted (Article 51 of the General Administrative Procedure Act) to determine the facts. *Data breach notification* is a notification that the controller needs to relay to the supervisory authority within 72 hours after finding out about data breach (Article 33 of GDPR), unless the personal data breach is unlikely to result in a risk to the rights and freedoms of natural persons. In Croatia the competent data protection authority is CPDPA.

Notification of a personal data breach to the supervisory authority was not an obligation according to the previous Directive 95/46/EC and Act on Personal Data Protection, so this is one of the provisions which represent a new obligation for data controllers. Therefore, there is no data for data protection breach notification for the period prior to the enforcement of the GDPR.

### Data collection

We obtained information from CPDPA about requests/complaints related to personal data protection that were received specifically from academic/research institutions, and the number and type of all cases/requests in years 2015-2019. Based on the date of the GDPR enforcement, data were further subdivided into cases received from January 1, 2015 to May 24th 2018 (pre-GDPR period) and cases received from May 25th 2018 to May 24th 2019 (post-GDPR period).

For cases/requests from academic/research institutions, the following data were analysed: number of cases, number of cases that were not further processed, number of legal actions raised based on those cases and their outcomes, number of cases for which additional explanations were requested by CPDPA in a query to the originator of the case, and number of responses to such requests. We categorized all cases.

Additionally, CPDPA provided the following anonymized information about all requests related to data protection in the analysed period: total number of cases/requests received. Approval of the research ethics committee for this study was not required, as the analysed data were completely anonymous.

### Statistical analysis

Data were reported as descriptive statistics, with frequencies.

## Results

### Requests from academic and research institutions

From January 2015 till the end 2019, CPDPA received only 37 requests about personal data protection related to the use of data for research purposes (Table 1). In the analysed pre-GDPR period, there were 21 requests sent to CPDPA by academic and research institutions from Croatia and in the post-GDPR period there were 16 such requests. The majority of requests (N = 36) were related to questions about data protection for research-related purposes, where only the opinion of CPDPA was sought. One inquiry asked CPDPA about the definition of scientific research used in Croatia.

**Table 1:** Categorized requests related to use of personal data in research received by Croatian Agency for personal data protection since until June 2019

**Date of query submission**	**Institution/person that submitted query**	**Category**	**Medical or non-medical research?**
December 2015	University Department	Complaint from a researcher that they were unable to obtain data from a public institution from Croatia, based on a prior agreement with that institution	Medical
May 2015	PhD student	Asking advice regarding collecting research data from minor persons that would be included in a study	Unclear
October 2015	University Department	Asking advice regarding contacting former and current students of several university programs, regarding participation in a survey within a European project	Non-medical
February 2015	University Department	Asking advice regarding contacting adults to participate in a research study	Unclear
July 2015	University Department	Asking advice regarding collecting research data from minor persons that would be included in a study	Unclear
March 2015	Ministry	Asking advice regarding collecting data about seniors in nursing homes for a research study	Non-medical
February 2015	Public institute	Asking advice about providing patient data to University researchers for a research study	Medical
July 2015	Researcher	Asking advice about analysis of personal data obtained via e-mails, for conducting research/survey within the Master Thesis	Unclear
April 2015	University Department	Asking advice regarding collecting research data from minor persons that would be included in a study	Unclear
September 2016	Researcher	Asking advice about data analysis about health without patient consent, for the research-related purposes	Medical
March 2016	Ministry	Asking advice about accessing data from the researchers’ registry to be used in a digital archive and repository	Non-medical
April 2016	Employee of a data protection department from a research organization in Switzerland	Asking advice about using personal contacts of patients from existing clinical trial databases, for the purpose of conducting a new research	Medical
May 2016	PhD student	Asking advice about collecting and analysing data via anonymous survey of consumers	Non-medical
September 2016	State administration office	Asking advice about using birth records data for preparation of a monography about demographics	Non-medical
January 2016	State agency	Asking advice about providing data to the State department of statistics for research about salary of employees	Non-medical
May 2016	Research institute	Asking advice about providing data to the State department of statistics for research about salary of employees	Non-medical
February 2016	Pharmacy	Asking advice about providing data to the State department of statistics for research about salary of employees	Non-medical
May 2017	Research institute	Asking advice about coding results of blood sampling analyses	Medical
March 2017	Ministry	Asking advice about providing personal data of PhD students to a research institute for a research study	Non-medical
February 2018	Student	Asking advice about transfer of pseudonymized data to researchers outside of European Union	Unclear
April 2018	School	Asking advice about sharing data from students’ e-Diary with research institute for a research study	Non-medical
**Post-GDPR (The EU’s General Data Protection Regulation)**			
June 2018	University Department	Asking advice about sharing data about students to national research team for the purpose of a research study	Non-medical
June 2018	Student	General request about how GDPR affects research activities	Unclear
July 2018	University Department	Asking advice about obtaining personal data of individuals from the registry of Agency of Croatian civil aviation	Non-medical
July 2018	Research institute	Asking advice about transfer of personal data of Croatian artists and architects to the USA, for research-related purposes	Non-medical
September 2018	Hospital	Asking advice about allowing non-hospital employees to access patient data for research-related purposes	Medical
October 2018	Centre for research	Asking advice about public presentation of employee data collected during research study	Non-medical
December 2018	Student	Asking advice about including participants in a survey-type research	Unclear
December 2018	Researcher	Asking about definition of scientific research in Croatia, and how is it regulated	Unclear
February 2019	Student	Asking advice about transfer of pseudonymized data to researchers outside of European Union	Unclear
March 2019	Researcher	Asking advice about conducting survey among university students as a part of PhD thesis	Unclear
April 2019	Trade Union	Asking advice about personal data processing in the EU funded project and compliance with the GDPR	Non-medical
May 2019	Accounting company	Asking advice about sharing data with the Croatian Bureau of Statistics for the purpose of a study about structure of salaries in 2018	Non-medical
June 2019	Researcher	Asking advice about personal data processing for scientific project in another EU country	Non-medical
June 2019	Researcher from private company	Asking advice about personal data processing and GDPR compliance in the framework of EU funded project	Non-medical
June 2019	University	Asking advice about GDPR compliance in the research competences of adults	Non-medical
November 2019	Researcher	Asking advice opinion about relation between GDPR and Croatian law on right to access information for the purpose of Master thesis	Non-medical
GDPR - General Data Protection Regulation.			

Table 1 presents more details about requests related to research purposes that were received by CPDPA in the analysed period. Among 37 such requests, there were 6 about medical research, 20 about non-medical research, while in another 11 requests it was not clear which one of those two types of research the subject was referring to. In the entire analysed period, the CPDPA did not receive from academic and research institutions any reports or requests about potential data breaches and none of the submitted data protection requests resulted in subsequent initiation of legal disputes.

### All cases/requests received in 2015-2019

In 2018, CPDPA had a dramatic increase in the number of requests in the post-GDPR period, compared to the pre-GDPR period of the same year (Table 2). In 2019, CPDPA received 2718 requests/complaints, less than in the year 2018. In 2018 CPDPA received 3464 requests/requests for legal advice sent by data subjects, data controllers and data processors. Furthermore, in 2018 CPDPA received 356 complaints (217 more than in 2017) seeking a determination of a violation of rights. The largest number of complaints and requests pertained to issues related to video surveillance, contracts with telecommunication companies, handling of employees’ personal data, handling of personal data in the enforcement proceedings, personal data processing in tendering procedures, public disclosure of personal data in media and on social networks, and disclosure of personal data to third parties in excessive scale.

**Table 2:** Number of requests and complaints received by Croatian personal data protection agency

**Year**	**2015**	**2016**	**2017**	**Entire 2018**	**2018** **January 1 – May 24** **(pre-GDPR)**	**2018** **May 25 – December 31** **(post-GDPR)**	**Entire 2019**
Number of requests/legal advice	613	604	850	3464	859	2605	1406
Number of complaints	537	417	524	1226	319	907	1312
Number of administrative procedures	142	142	139	356	45	311	190
Number of data breach notifications	-	-	-	-	-	49	72
GDPR - General Data Protection Regulation.							

## Discussion

Our study indicates that the CPDPA received very few requests related to personal data protection from academic and research institutions in Croatia, both before and after enforcement of GDPR, and none of those requests was about a potential data breach. This was in stark contrast with the increase of number of general requests and non-research-related data breach cases that were received by the same Agency.

The number of requests submitted to CPDPA continued to increase in the analysed period, particularly after the full implementation of GDPR. An increase in the number of complaints shows that citizens are much more aware of their rights in relation to personal data protection, although in many cases they misinterpret their rights and for that reason there is no valid ground to initiate administrative procedures.

Since the number of requests to the national authority regarding personal data protection by academic and research institutions in Croatia was exceedingly low, we can provide several possible assumptions for that result. It could be possible that the data protection system in academic and research institutions functions perfectly, and that every researcher is familiar with GDPR having no need for further information and clarifications. However, knowing that on the national level a specific GDPR derogation for the use of personal data for research purposes except those used for the national statistics purposes are not foreseen, we consider that this is not very likely (*5*).

Another option is that GDPR is perfectly clear, and that researchers have no issues with it. Nevertheless, it has already been pointed out that GDPR may not be perfectly clear about what the researchers are supposed to do in certain situations. For example, when researchers collect data, GDPR stipulates that the processing of those data for purposes other than those for which the data were originally collected should be allowed only if the new purpose is compatible with the initial purpose of data collection. However, it has been highlighted by Orel *et al.* that it is not clear whether this presumed compatibility is completely automatic and whether researchers have to ensure additional requirements such as related to the data minimization principle (*7*).

Additionally, there are questions regarding participants’ informed consent. For example, Orel *et al.* highlighted there is a debate about the risk that GDPR will require consent of participants before each and every act of data processing, which was not envisioned in the initial study protocol; the question is now whether a broad consent of participants is sufficient and appropriate when there could be sensitive data involved, and which researchers could use later on for different and initially unknown and unplanned research purposes (*7*).

Another grey area for researchers, it has been reported, could be pseudonymization. In 2018 McCall warned that researchers with lack of knowledge about differences between pseudonymization and anonymization could consider that they are collecting anonymous data, which falls outside of the scope of GDPR (*8*). Data that are pseudonymized do count as personal data under GDPR, but a there are many technical and statistical measures that can be taken to make re-identification of individuals when using pseudonymization very difficult. However, Kohlmayer *et al.* highlighted that this increased complexity could even lead to new attack vectors for intruders, which would be in stark contrast to the primary objective of improving personal data protection (*9*).

In their 2019 article, Shabani *et al.* have described particular challenges with GDPR in terms of genomic data, because with the narrow focus on an individual, the GDPR has neglected issues and concerns that affect a collectivity (*10*). For example, Lin *et al.* have demonstrated that few single nucleotide polymorphisms (SNPs) are sufficient to distinguish a DNA record of an individual (*11*). Furthermore, there are open-access platforms with genomic data, and genetic and genomic data convey information not only about a single individual, but also about their relatives and ethnic heritage (*12*, *13*). The most likely explanation for our data is that there is low awareness in academic and research institutions in Croatia about the implications of GDPR, and specifically about the lack of implementation of crucial aspects of GDPR into Croatian law regarding data protection and privacy issues related to research and innovation. However, this is only an assumption, as we were unable to find studies in the research literature about the awareness of researchers about GDPR.

We came across a survey that showed GDPR awareness index among consumers was low, and a survey reporting that the majority of organizations failed to comply with the May 2018 deadline to comply with GDPR and that one year after the GDPR implementation compliance to GDPR was still a challenge (*14*, *15*). Potential lack of knowledge about personal data protection among researchers may have legal implications, as well as implications for applications for funding. For example, Croatia has not been very successful in attracting funding from the European Commission’s H2020 research programme compared to some other European countries (*16*). Such projects tenders have very strict rules for cases when collection and processing of personal data are used. Therefore, theoretically it is possible that applicants not demonstrating high level of awareness of ethics issues, which may arise from the use of personal data when used for research purposes, may be less successful in such applications. Given the recent advent of GDPR, and its mandatory nature for EU researchers, it would be worthwhile now to start exploring whether researchers are aware of it, what are the challenges for implementing it, and whether academic and research institutions provide adequate support to researchers for GDPR compliance.

It is still unknown what role data protection officers (DPO) are fulfilling in research institutions, whether and to what degree their help was required both from the side of researchers and institutional ethics committees that grant approvals for research involving collection and processing of personal data protection. That is why the quality of DPOs fulfilment of their roles in research and academic institutions remains fully uncertain. Even though it is highly recommended that the DPOs should continuously develop their knowledge and skills in the demanding area of personal data protection, there is no specific legal obligation about that on the level of GDPR or national law. Article 37, recital 5, clearly states that the data protection officer shall be designated on the basis of professional qualities and, in particular, expert knowledge of data protection law and practices (*3*). Importantly, there is no specific legal obligation according to which institutional Ethics Committees would need to consult DPOs for the case that they are granting approvals for research involving collection and processing of personal data. If the institution conducting the scientific research has appointed a DPO, it is important to include him/her in all stages of the research and to seek advice on all issues related to personal data protection. If not appointed, the advice of the relevant expert should be sought already at the stage of preparation of the project proposal. Furthermore, there are no data about mechanisms of appointing DPOs, their knowledge and education about data protection and privacy, and whether they are aware of their pivotal data consultancy role for some research involving personal data collection and processing in their institutions.

In conclusion, very few requests about personal data protection from academic and research institutions in Croatia were submitted to the national Croatian data protection authority. Future studies could explore whether researchers have sufficient awareness and knowledge about personal data protection related to research, to adequately implement the GDPR regulations. In case that future studies confirm insufficient awareness of GDPR regulations and requirements among relevant stakeholders, interventions both on the national and EU level will be needed to rectify this.
